# Seminal Plasma as a Source of Prostate Cancer Peptide Biomarker Candidates for Detection of Indolent and Advanced Disease

**DOI:** 10.1371/journal.pone.0067514

**Published:** 2013-06-24

**Authors:** Jochen Neuhaus, Eric Schiffer, Philine von Wilcke, Hartwig W. Bauer, Hing Leung, Justyna Siwy, Wolfram Ulrici, Uwe Paasch, Lars-Christian Horn, Jens-Uwe Stolzenburg

**Affiliations:** 1 University of Leipzig, Department of Urology, Leipzig, Germany; 2 Mosaiques Diagnostics GmbH, Hannover, Germany; 3 Ludwig-Maximilians-Universität, Urology Maximilianstrasse, Munich, Germany; 4 Beatson Institute for Cancer Research, Glasgow, United Kingdom; 5 Medical Practice, Leipzig, Germany; 6 University of Leipzig, Department of Dermatology, Leipzig, Germany; 7 University of Leipzig, Department of Pathology, Leipzig, Germany; Biomedical Research Foundation, Academy of Athens, Greece

## Abstract

**Background:**

Extensive prostate specific antigen screening for prostate cancer generates a high number of unnecessary biopsies and over-treatment due to insufficient differentiation between indolent and aggressive tumours. We hypothesized that seminal plasma is a robust source of novel prostate cancer (PCa) biomarkers with the potential to improve primary diagnosis of and to distinguish advanced from indolent disease.

**Methodology/Principal Findings:**

In an open-label case/control study 125 patients (70 PCa, 21 benign prostate hyperplasia, 25 chronic prostatitis, 9 healthy controls) were enrolled in 3 centres. Biomarker panels a) for PCa diagnosis (comparison of PCa patients versus benign controls) and b) for advanced disease (comparison of patients with post surgery Gleason score <7 versus Gleason score >7) were sought. Independent cohorts were used for proteomic biomarker discovery and testing the performance of the identified biomarker profiles. Seminal plasma was profiled using capillary electrophoresis mass spectrometry. Pre-analytical stability and analytical precision of the proteome analysis were determined. Support vector machine learning was used for classification. Stepwise application of two biomarker signatures with 21 and 5 biomarkers provided 83% sensitivity and 67% specificity for PCa detection in a test set of samples. A panel of 11 biomarkers for advanced disease discriminated between patients with Gleason score 7 and organ-confined (<pT3a) or advanced (≥pT3a) disease with 80% sensitivity and 82% specificity in a preliminary validation setting. Seminal profiles showed excellent pre-analytical stability. Eight biomarkers were identified as fragments of N-acetyllactosaminide beta-1,3-N-acetylglucosaminyltransferase, prostatic acid phosphatase, stabilin-2, GTPase IMAP family member 6, semenogelin-1 and -2. Restricted sample size was the major limitation of the study.

**Conclusions/Significance:**

Seminal plasma represents a robust source of potential peptide makers for primary PCa diagnosis. Our findings warrant further prospective validation to confirm the diagnostic potential of identified seminal biomarker candidates.

## Introduction

Prostate cancer (PCa) is the second most frequently diagnosed cancer and the sixth leading cause of cancer death in males worldwide [1]. The introduction of serum prostate specific antigen (PSA) screening led to a significant increase in the number of diagnosed cases [2] but failed to demonstrate a statistically significant prostate cancer mortality benefit [3]. Ninety-five percent of men with PSA-detected cancer who are followed for 12 years do not die from PCa, even in the absence of definite treatment, such as radical prostatectomy, radiation therapy or hormonal therapy [3].

This has significantly exaggerated our current inability to make evidence-based recommendations on treatment choices according to tumour behaviour, namely clinically insignificant, or indolent disease and clinically significant, or advanced disease [4]. Therefore, new screening modalities are urgently needed to reduce the number of men who require biopsy and to improve the discriminatory accuracy between indolent tumour that has a favourable clinical prognosis even without intervention, and disease that is likely to have already clinically advanced, in order to reduce over-diagnosis and over-treatment.

Proteomic biomarker screening has become popular during the past decade. Blood, urine, prostatic fluids, and prostatic tissue have been evaluated as biomarker source. Several candidate biomarkers found in those studies were introduced as biomarkers in an attempt to address the clinical needs for discrimination of indolent and advanced disease [5]-[7]. However, all the single biomarkers currently available, lack diagnostic accuracy for routine clinical application. The high biological variability of prostate cancer suggests that a distinct clearly defined set of biomarkers, rather than a single biomarker, may be more efficient to accurately assess the disease. Recent technical advances, especially in mass spectrometry and computation, allow application of proteomic profiling for discovery of multiple protein biomarker.

Recently, we identified and validated a proteomic pattern of 12 naturally occurring, urinary peptide biomarkers by capillary electrophoresis mass spectrometry (CE-MS), capable to detect PCa using first stream urine with 90% sensitivity and 61% specificity [8], [9]. These experiments suggested that prostatic fluids may serve as source of biomarkers [10]. On the basis of these findings, we hypothesized that seminal plasma might offer a robust source to identify novel PCa protein maker profiles. This study aimed at a systematic assessment of pre-analytical seminal plasma stability and of its suitability for the development of PCa biomarker panels.

## Results

### Patients’ clinical outcome

**Figure 1:**
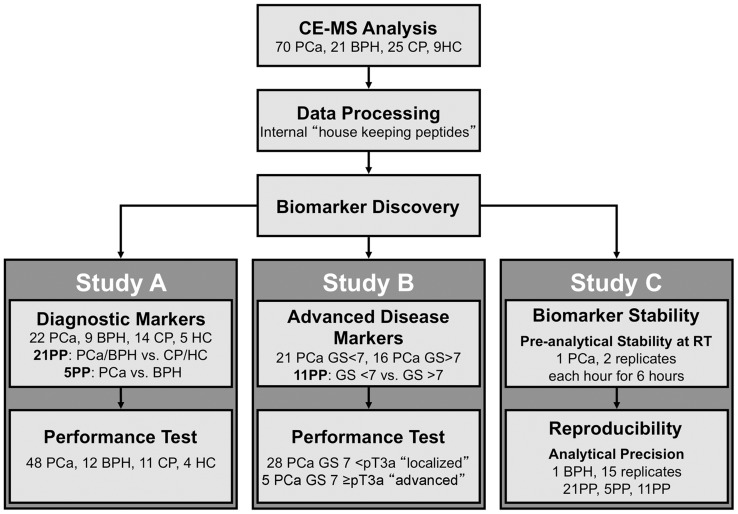
Flow chart of study design. For biomarker discovery in total 125 seminal plasma samples were used from 70 patients with PCa, 21 patients with benign prostate hyperplasia (BPH), 25 patients with chronic prostatitis (CP) and 9 healthy control (HC). This pool of available samples was used in varying composition in three study arms. In study A “Diagnostic Markers” 50/125 patients with and without prostate cancer (22 PCa, 14 CP; 9 BPH and 5 HC) were used for biomarker discovery and the remaining 75/125 patients (48 PCa, 12 BPH, 11 CP, and 4HC) were used for diagnostic performance tests. In Study B “Advanced Disease Markers” available PCa samples (n = 70) were stratified according to Gleason score. For biomarker discovery patients with Gleason score <7 (n = 21) and Gleason score >7 (n = 16) were compared. The remaining 33/70 patients with Gleason score 7 (28 indolent disease <pT3a and 5 ≥pT3a advanced disease according EUA guidelines) were used for testing clinical performance. Furthermore, in study C preliminary assessment of stability and precision of the approach was performed.

In total 70 patients with PCa, 21 patients with benign prostate hyperplasia (BPH), 25 patients with chronic prostatitis (CP) and 9 healthy control (HC) were included in the study (Table 1 and Figure 1). CP and HC groups were significantly younger than the patients in the PCa and the benign prostate hyperplasia (BPH) groups (Table 1). As expected PSA levels were significantly lower in CP and HC compared to BPH (0.98 – 6.70 ng/ml) or PCa (2.0 – 20 ng/ml) in both, training and test set (p<0.05, Mann Whitney test, two-tailed; Table 1). The TNM classification revealed 60 organ confined (≤pT2c) and 10 advanced (≥pT3a) PCa. The allocation of patients to low and high risk groups varied considerably between classification systems (Table 1).

**Table 1:** Patient descriptive statistics.

A	Training set									
Group	N	Age (95%CI)	PSA [ng/ml] (95%CI)	Gleason sum (N)	Histology (N)	D'Amico/AUA	NCCN	EAU	RTOG	CAPRA
CP	14	39.79±13.58 (31.94-47.63) § $	1.017±0.7265 (0.4582-1.575) $	n.a.	n.a.	n.a.	n.a.	n.a.	n.a.	n.a.
HC	5	42.40±9.34 (30.80-54.00) §	1.202±0.6499 (0.3951-2.009) $	n.a.	n.a.	n.a.	n.a.	n.a.	n.a.	n.a.
BPH	9	59.56±6.064 (54.89-64.22)	3.824±1.839 (2.411-5.238)	n.a.	n.a.	n.a.	n.a.	n.a.	n.a.	n.a.
PCa	22	57.86±6.882 (54.81-60.91)	7.611±4.138 (5.777-9.446)	≤6 (5) 7 (10) >7 (7)	pT2a, G1 (1) pT2c, G2 (5) <pT3, G3 (11) pT3a, G3 (4) pT3b, G3 (1)	low risk (1) intermed. (2) high risk (19)	low risk (11) high risk (11)	low risk (16) high risk (6)	low risk (20) high risk (2)	low risk (1) intermed. (16) high risk (5)
**B**	**Test set**									
CP	11	49.82±14.48 (40.09-59.54) $	1.952±1.548(0.8448-3.059) $	n.a.	n.a.	n.a.	n.a.	n.a.	n.a.	n.a.
HC	4	53.75±12.04 (34.59-72.91) $	0.855±0.6035 (-0.1052-1.815) $	n.a.	n.a.	n.a.	n.a.	n.a.	n.a.	n.a.
BPH	12	62.00±5.187 (58.70-65.30)	5.486±2.253 (4.055-6.917)	n.a.	n.a.	n.a.	n.a.	n.a.	n.a.	n.a.
PCa	48	59.46±6.776 (57.49-61.43)	8.362±4.088 (7.175-9.549)	≤6 (16)7 (23)>7 (9)	≤pT2c, G2 (19)≤pT2c, G3 (24) pT3a, G2 (1) ≥pT3a, G3 (4)	low risk (2) intermed. (7) high risk (39)	low risk (32) high risk (16)	low risk (40) high risk (8)	low risk (38) high risk (10)	low risk (3) intermed. (26) high risk (13)

CP  =  chronic prostatitis; HC  =  healthy control; BPH  =  benign prostata hyperplasia; PCa  =  prostate carcinoma; n.a.  =  not applicable or not available; §  =  sign. vs. BPH; $  =  sign. vs. PCa (two-tailed Kruskal-Wallis parameter free ANOVA with Dunn’s Multiple Comparison Test); D’Amico [28] adopted by the AUA  =  American Urology Association [47]; NCCN  =  National Comprehensive Cancer Network [29]; EAU  =  European Association of Urology [31]; RTOG  =  Radiation Therapy Oncology Group [30]; CAPRA  =  Cancer of the Prostate Risk Assessment Score [48].

### Proteomic profiles

**Figure 2:**
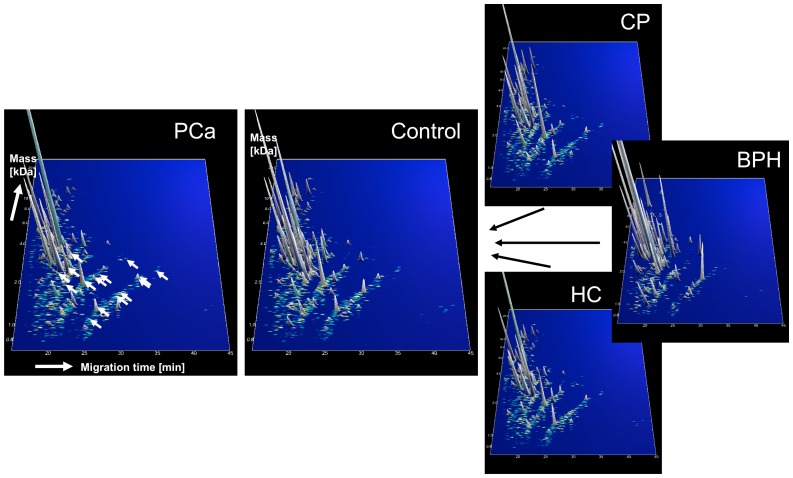
Human seminal plasma polypeptide profiles. Capillary electrophoresis coupled to mass spectrometry profiling of humane seminal plasma revealed a total of 1784 peptides. The synthetic peptides spiked to the samples for pre-calibration purposes are marked with white arrows. Normalized molecular weight (700-25.000 Da) in logarithmic scale is plotted against normalized migration time (15-45 min). The mean signal intensity of the polypeptide peak is given in 3D-depiction. Compiled data sets of PCa (case) combined all controls and also separately CP (control), BPH (control) and HC (control) from training set are shown.

CE-MS analysis yielded high resolution profiles (Figure 2, Table S1). For preliminary profile calibration we used synthetic isotope labelled peptides as reference. This pre-calibration allowed definition of 287 “house-keeping peptides” as reference mass and migration time data points. As ion signal intensity (amplitude) showed significant variability, the signals of 46 highly abundant peptides were used as internal standard peptides for signal normalization (Table S2). These peptides were present in >97% of analyzed samples and showed lowest signal variability. The procedure to use “internal standard” for amplitude normalization, was shown to be an easy and reliable method to address both analytical and dilution variances in a single calibration step [11]. Tandem mass spectrometry [12]-[14] identified 141 native seminal peptides representing 47 different parental proteins (Table S3). Eighty-eight identified peptides (83/141, 59%) were fragments of semenogelin-1 or -2, by far the most abundant peptides of the low molecular weight seminal proteome.

### Biomarker discovery

#### Study A: Diagnostic markers

**Figure 3:**
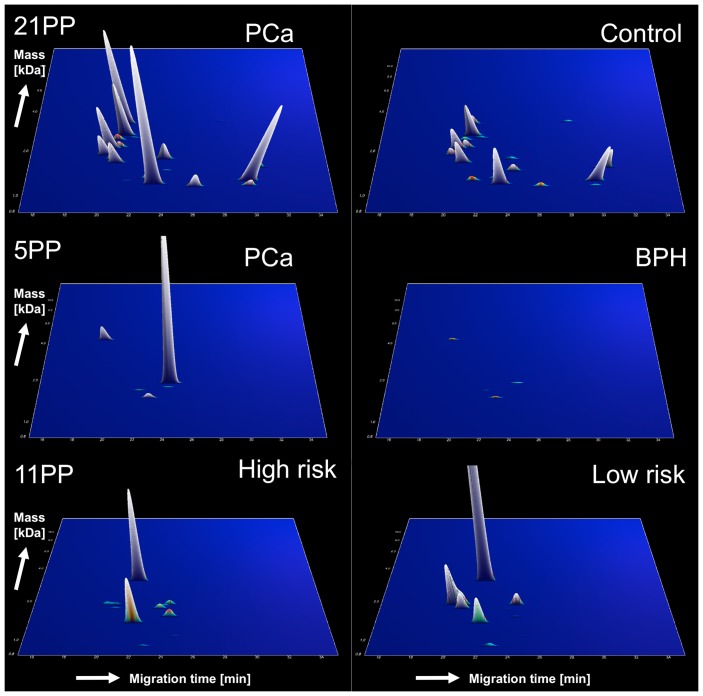
Biomarker signatures. Normalized molecular weight (700–25.000 Da) in logarithmic scale is plotted against normalized migration time (15–45 min). The mean signal intensity of the polypeptide peak is given in 3D-depiction. Averaged data sets of the training set are shown.

For diagnostic biomarker discovery we divided the available 125 samples into a discovery set with 22 PCa, 14 CP; 9 BPH and 5 HC samples and the remaining 48 PCa, 12 BPH, 11 CP, and 4HC samples into an independent test set (Figure 1). Multiple testing statistics resulted in 21 discriminatory polypeptides significantly altered between patients with and without prostate cancer (Table 2 and Figure 3). Six out of the 21 polypeptides were identified as fragments of N-acetyllactosaminide beta-1,3-N-acetylglucosaminyltransferase, prostatic acid phosphatase, semenogelin-1 and -2 (Table 3). In order to define biomarker candidates reliably differentiating PCa and BPH, we compared BPH *vs.* PCA, BPH *vs.* CP & HC, and BPH *vs.* PCa & CP & HC using appropriate multiple testing statistics. Five polypeptides were significant in all three tests suggesting suitability of these candidates to specifically identify BPH and therefore to exclude presence of PCa (Table 2, Figure 3). One of them was a fragment of GTPase IMAP family member 6 (Table 3).

**Table 2:** Polypeptides constituting the biomarker signatures 21PP, 5PP, and 11PP, respectively.

Peptide ID	Mass [Da]	CE-time [min]	Mean PCa	Mean BPH/HC	Regulation+	P value (Wilcoxon)	P value (BH)
3495	1173.56	25.9	38	100	-2.7	0.0012	0.0926
3506	1174.55	26.1	544	77	7.1	0.0137	0.0389
3621	1186.45	29.6	281	41	6.8	0.0022	0.0926
3992	1217.60	23.4	3770	1412	2.7	0.0021	0.0926
4437	1254.56	23.5	92	17	5.3	0.0022	0.0926
4679	1277.56	29.6	2600	930	2.8	0.0021	0.0926
4697	1279.56	21.6	25	252	–10.2	0.0023	0.0928
5180	1328.64	23.1	299	115	2.6	0.0024	0.0962
6832	1488.80	24.2	46	280	–6.0	0.0023	0.0928
7661	1576.80	30.3	260	911	–3.5	0.0023	0.0928
8698	1691.76	20.9	853	1648	–1.9	0.0022	0.0926
9483#	1779.92	20.7	398	470	–1.2	0.0023	0.0928
9645	1797.95	24.1	644	43	14.9	0.0025	0.0926
10502#	1917.92	20.1	798	1385	–1.7	0.0023	0.0928
11899	2139.08	20.4	1501	2749	–1.8	0.0024	0.0977
12083	2167.12	21.1	257	516	–2.0	0.0022	0.0926
13995	2461.29	20.9	243	282	–1.2	0.0022	0.0926
14592	2556.29	21.9	34	158	–4.6	0.0023	0.0928
15331	2670.40	21.4	1742	6996	–4.0	0.0022	0.0926
18990	3266.65	21.4	2645	451	5.9	0.0021	0.0926
19773	3400.52	28.1	10	89	–8.8	0.0021	0.0926

Peptide ID	Mass [Da]	CE-time [min]	Mean BPH	Mean PCa/HC	Regulation	P value (Wilcoxon)	P value (BH)
7098	1515.83	22.9	872	2853	–3.3	0.0004	0.0048
8863	1709.85	22.2	62	481	–7.7	<0.0001	0.0012
9673	1801.95	24.3	19	1378	–73.1	0.0013	0.0022
10706	1948.06	24.4	571	71177	–124.8	0.0020	0.0251
24050	4385.19	19.4	585	6498	–11.1	0.0027	0.0345

Peptide ID	Mass [Da]	CE-time [min]	Mean High risk	Mean Low risk	Regulation	P value (Wilcoxon)	P value (BH)
1004	929.48	22.8	47	131	–2.8	0.0390	N.A.
2663	1097.56	24.8	34	7	4.8	0.0188	N.A.
5650	1372.71	22.0	2029	1206	1.7	0.0448	N.A.
7228	1531.81	24.4	375	37	10.2	0.0363	N.A.
9483#	1779.92	20.7	78	758	–9.8	0.0318	N.A.
9504	1782.84	23.8	233	27	8.5	0.0124	N.A.
10193	1869.86	24.1	75	3	29.4	0.0005	N.A.
10442	1908.97	20.7	101	614	–6.1	0.0202	N.A.
10471	1912.97	24.4	221	569	–2.6	0.0258	N.A.
10502#	1917.92	20.1	143	1814	–12.7	0.0316	N.A.
17993	3100.56	22.1	3629	8420	–2.3	0.0392	N.A.

ID: polypeptide identifier annotated by the SQL database (ID).

+ : upregulated biomarkers: mean(case)/mean(control);

downregulated biomarkers: -mean(control)/mean(case).

# : Biomarker of 21PP and 11PP.

**Table 3:** Biomarker sequence data.

Peptide ID	Masse [Da]	CE-time [min]	Sequence	Protein name	UniProt ID	start AA	stop AA	Theo. Mass [Da]	▵M[ppm]	Regulation
3506	1174.55	26.1	LLAALMLVAmL	N-acetyllactosaminide beta-1,3-N-acetylglucosaminyltransferase	O43505	15	25	1174.575	–21	PCa up
4679	1277.56	29.6	TELYFEKGEY	Prostatic acid phosphatase	P15309	316	325	1277.582	–17	PCa up
5650	1372.71	22.0	LPNLLMRLEQm	Stabilin-2	Q8WWQ8	1137	1147	1372.721	–4	High risk up
7098	1515.83	22.9	LSAPGPHAVLLVTQL	GTPase IMAP family member 6	Q6P9H5	118	132	1515.817	9	BPH down
11899	2139.08	20.4	TEELVANKQQRETKNSHQ	Semenogelin-1	P04279	198	215	2139.067	6	PCa down
12083	2167.12	21.1	TEELVVNKQQRETKNSHQ	Semenogelin-2	Q02383	198	215	2167.098	10	PCa down
15331	2670.40	21.4	YVLQTEELVVNKQQRETKNSHQ	Semenogelin-2	Q02383	194	215	2670.373	10	PCa down
18990	3266.65	21.4	SQTEEKAQGKSQKQITIPSQEQEHSQKAN	Semenogelin-1	P04279	316	344	3266.613	11	PCa up

ID: polypeptide identifier annotated by the SQL database (ID); Theo. Mass: theoretical mass of the peptide sequence; ▵M: Mass difference between experimental and theoretical mass normalized to theoretical mass in parts per million [ppm]. m: oxidized Methionine.

We applied a two-step approach: (i) a first panel (21 polypeptides, 21PP) to discern PCa and BPH from inflammatory and healthy prostate; (ii) a second panel (5 polypeptides, 5PP) to differentiate PCa and BPH. Both signatures were trained in the discovery cohort (Figure 1) using support vector machine algorithms (SVM) and reached AUC values of 100% (95% CI 93%–100%) for 21PP and 99% (95% CI 90%–99%) for 5PP.

For confirmation of classification performance of the biomarker signatures we applied the combination of 21PP and 5PP to an independent test set of 48 PCa, 12 BPH, 11 CP, and 4HC (Figure 1). Samples positive for 21PP (above the classification cut off) were re-classified using 5PP to specifically identify BPH excluding PCa. Therefore, samples positive for 21PP and negative for 5PP were considered as PCa, samples positive in either panels were considered as BPH and samples negative for 21PP (below the classification cut off) were considered as CP or HC control samples. This approach correctly identified 40 out of 48 PCa samples [83% sensitivity (95% CI 70%–93%)], 6 of 12 BPH and 12 of 15 controls [67% specificity (95% CI 46%–83%)]. AUC value was 75% (95% CI 64%–83%, *P* = 0.0001). The observed diagnostic performance was as high as the performance of PSA as reference, which showed 87% sensitivity (95% CI 75%–97%) and 59% specificity (95% CI 40%–80%).

#### Study B: Advanced disease biomarkers

For advanced disease biomarker discovery we divided the available 70 PCa samples into a training set with 37 PCa samples (21 post-surgery Gleason score <7, 16 post-surgery Gleason score >7). The remaining 33 samples with post-surgery Gleason score 7 were used as a test set. Comparison of the 21 GS <7 patients (<pT3a) to 16 GS >7 patients (11 <pT3a, 5 pT3a) using statistics corrected for multiple testing resulted in 11 biomarker candidates with a fragment of stabilin-2 among them (Tables 2 and 3). These as pattern (11PP) were found to classify the cohort with an AUC of 99% (95% CI 87%–100%, Figure 1).

To test the performance of the biomarkers associated with advanced disease, 11PP was applied to the test set of patients with post-surgery Gleason score 7 that were not used for biomarker discovery. Of the 33 samples, 9 scored as advanced (above the classification cut off) and 24 as indolent tumour (below the classification cut off).

**Figure 4:**
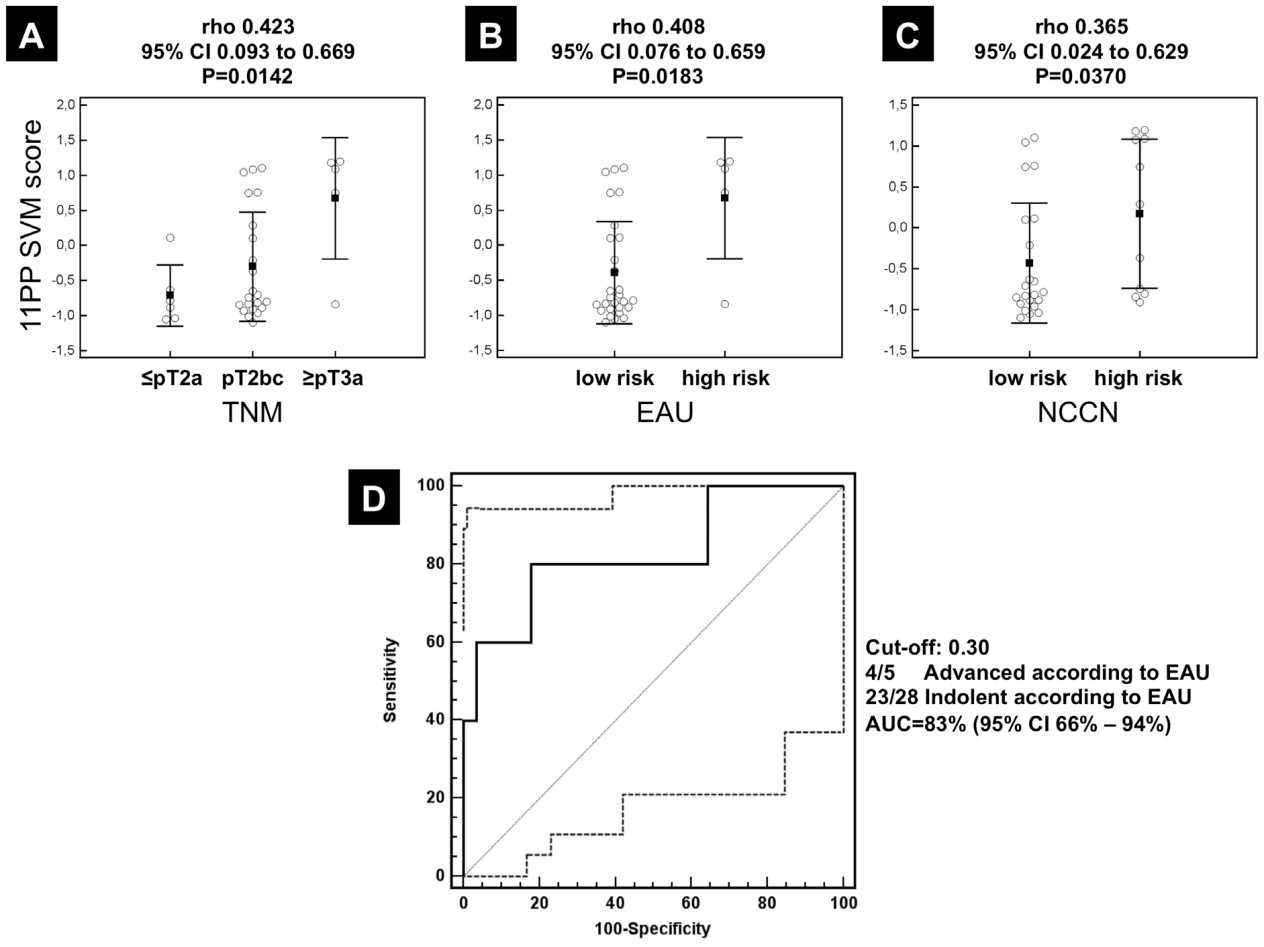
Biomarker performance validation. (**A**).Box and whisker plots of obtained 11PP results in the test cohort of PCa patients with GS 7 stratified according to TNM, (**B**) EAU, and (**C**) NCCN classification systems. Black squares indicate medians and whiskers 1.5-times the interquartile ranges. Rank correlation coefficients rho, the respective 95% CI and P-values are given above. (**D**) ROC curve (black lines) for 11PP classification of the independent validation cohort of PCa patients with GS 7 with either indolent (N = 28) or advanced (N = 5) disease according to EAU classification as reference standard. 95% confidence intervals are plotted as dashed lines. Diagonal line represents guessing probability with an area under the curve of 0.5. 95% confidence intervals (CI) are displayed as dashed lines.

**Figure 5:**
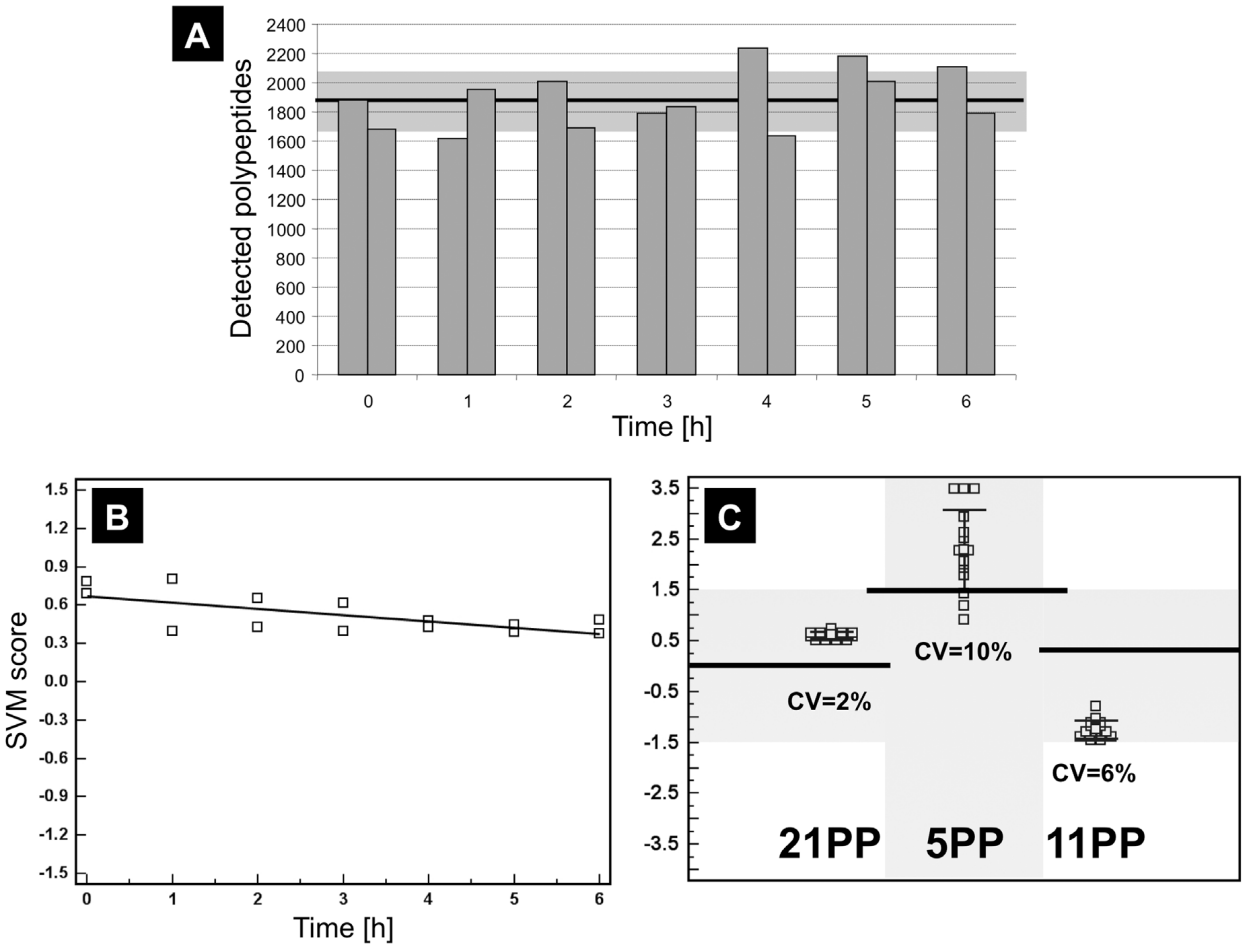
Assessment of biomarker stability and reproducibility. (**A**) An average of 1887±202 polypeptides was detected in each of the 14 measurements stored for different times at RT. The mean is marked with a bold line; standard deviation is highlighted in grey. (**B**) Beyond this qualitative assessment biomarker signatures were applied to the 14 stability replicates to obtain quantitative data of time-dependent stability of seminal plasma. For 21PP ranked correlation analysis revealed a significant decrease of SVM scores over time with Spearman’s rho of –0.576 (95% CI –0.854 to –0.07, P = 0.0379). Regression analysis unveiled a decrease rate of –0.05 a.u. (<2%) per hour. 5PP and 11PP displayed no significant time dependency. (**C**) Analytical precision of the established SVM classifiers was assessed by applying it to 15 CE-MS data sets obtained from independent replicates of a sample of a 57 years old patient with significant BPH. Mean classification scores were 0.619±0.07, 2.290±0.81, and -1.239±0.18 for 21PP, 5PP, and 11PP respectively. Coefficients of variations were calculated by dividing standard deviations by the observed overall range of SVM scores [highlighted in grey, 21PP from –1.50 to +1.50 (3.0 a.u.), 5PP from 4.50 to +3.0 (7.5 a.u.), and 11PP from –1.50 to +1.50 (3.0 a.u.)]. Coefficients of variations were 2.2%, 10.8%, and 6.1%, respectively. Classification cut offs are represented by horizontal lines. The boxes depict means and standard deviation as whiskers.

In clinical practice various classification systems are used to estimate risk for prostate cancer progression. Therefore, we compared the performance of our biomarkers to five commonly used systems (Table S4): 11PP results were significantly correlated to TNM stages [rho 0.423 (95% CI 0.093 to 0.669), P = 0.0142], EAU score [rho 0.408 (95% CI 0.076 to 0.659), P = 0.0183], and NCCN score [rho 0.365 (95% CI 0.024 to 0.629), P = 0.0370] (Figure 4A–C), while CAPRA, RTOG and D’Amico score were not correlated (data not shown). Using EAU classification as reference standard, 11PP correctly identified 4/5 advanced (≥pT3a) and 23/28 organ-confined (<pT3a) tumours, resulting in an AUC of 83% (95% CI 66%–94%, *P* = 0.0055, two sided power β = 0.84, Figure 5D). Sensitivity was 80% (95% CI 29%–97%) and specificity was 82% (95% CI 63%–94%)].

#### Study C: Assessment of biomarker stability and reproducibility

Seminal plasma demonstrated robust pre-analytical stability at room temperature. The obtained profiles were highly similar without massive disappearance or formation of degraded fragments. An average of 1887±202 peptides (Figure 5A) was detected in 14 replicates. Investigation of the 21PP in these 14 replicates to quantify time dependency of stability revealed a significant decrease of SVM scores over time with Spearman’s rho of –0.576 (95% CI –0.854 to –0.07, P = 0.0379, Figure 5B). Regression analysis unveiled a decrease rate of -0.05 a.u. (<2%) per hour. 5PP and 11PP displayed no significant time dependency. Analytical precision of the established SVM classifiers was assessed in 15 independent replicates. Mean classification scores were 0.619±0.07, 2.290±0.81, and -1.239±0.18 resulting in coefficients of variations of 2.2%, 10.8%, and 6.1% for 21PP, 5PP, and 11PP, respectively (Figure 5C).

## Discussion

We hypothesized that seminal plasma is a robust source of novel PCa peptide maker profiles with the potential to improve primary diagnosis of prostate cancer and to distinguish advanced from indolent disease.

In contrast to earlier reports of proteomic profiling of seminal plasma using tryptic digestion [15], we used native seminal plasma for biomarker proteomic analysis. The main advantages of this top-down approach on naturally occurring peptides include the ability to directly detect combinations of post-translational modifications, sequence variants, and degradation products. We detected almost 2,000 different seminal peptides ≤20 kDa. Those were fragments of larger parental proteins, which were partially also detected earlier using tryptic digests. However, our approach also identify yet unknown seminal constituents (Table S3).

The generation of these naturally occurring peptides depends on the proteolytic liquefaction of the ejaculate and results in multiple proteolytic fragments of seminal proteins. Disease associated alterations in this proteolytic liquefaction process might account for our observation, that some naturally occurring fragments show significantly altered seminal levels and others of the same parental protein do not. Therefore, pre-analytical stability and analytical reproducibility are of utmost importance for successful biomarker discovery and clinical validation. A first milestone in the current study was the development of a simple and reproducible sampling procedure consistent with a clinical routine setting. We allowed liquefaction to reach a final steady state, documented by a constant number of detectable polypeptides over time (Figure 5A), but controlled time to sample storage at –80°C to be below 60 min to avoid interference with time-dependent biomarker instability at room temperature (Figure 5B).

Samples of prostate tissue, blood, seminal plasma, and urine with and without prostate massage are currently intensively analyzed for potential PCa biomarkers [5], [6]. While tissue is expected to be proximal to the origin of the disease and to correlate with highest biomarker concentrations, the sampling of tissue is related to invasive intervention with all risks and limitations. In contrast, especially seminal plasma and urine are easily accessible. However, proteolytic processing is of increasing importance for the exploitation of markers from bodyfluids. Our preliminary data on seminal plasma stability (Figure 5A/B) did not provide evidence for massive post-sampling degradation as in contrast was observed for blood serum [16] or plasma [17]. Therefore seminal plasma might combine high proximity to the prostate gland as site of the tumour only exceeded by direct prostate tissue sampling with the excellent stability and accessibility of urine [18]–[21], making it a highly promising source for potential PCa biomarkers.

We were able to define and validate robust biomarker signatures for the diagnosis of PCa. The sensitivity of 83% (95% CI 70%–93%) to diagnose PCa was highly comparable with those reported earlier for CE-MS based urinary biomarker signatures (sensitivity 86% to 90%). The specificity of 67% (95% CI 46%–83%) was slightly better than their urinary counterparts of 59% and 61%, respectively [8], [9].

In addition, we discovered a seminal biomarker signature, which distinguished (*P* = 0.0055) patients with post-surgery Gleason score 7 with indolent (<pT3a) or advanced (≥pT3a) disease with high sensitivity and specificity of 80% and 82%, respectively. Current clinical routine using serum PSA level and pre-surgery Gleason sum score to identify advanced disease remains inadequate, as the majority of screening detected PCa have PSA levels between 4–10 ng/ml and moderate Gleason sum scores of 6 and 7. Therefore, these biomarkers, which are based on post-surgery outcome data as reference standard, might represent a future possibility for a non-invasive pre-surgery differentiation of organ confined and advanced tumour stages. In addition, tumour evaluation by pre-surgery Gleason score grading requires invasive procedures to obtain tissue specimens, and is hampered by significant inter-operator variability and discrepancies between pre- and post-surgery scores in as many as 35% of cases [22]. Furthermore, among patients with clinically localised disease (tumour stages T1 and T2), approximately 30% are found to have locally advanced tumours following radical surgery. Therefore, there is a real risk of under-treatment in this group of patients, if managed by surveillance. In future the biomarker profile might help to avoid under-treatment in these patients with unclear clinical presentation.

One of the differentially expressed seminal proteins was prostate acidic phosphatase (ACPP), which is a negative regulator of cell growth in LNCap cells [23]. Down regulation of cellular ACPP is associated with androgen-independent tumour growth and high tumorigenicity of advanced PCa grades [23].

We observed semenogelin-1 fragment 316–344 (ID18990) as one of the 21 differentially regulated polypeptides (Table 2 and Table 3). While this fragment can directly be assigned to KLK3 ( = PSA) cleavage at site 315 (SSIY-SQTE), this holds not true for the other observed semenogelin fragments. These cannot be explained by KLK3 cleavage alone, implicating presence of a more complex protease activity network with multiple downstream cleavage events after initial KLK3 cleavage It is well known that there are mutual activation and inhibition mechanisms within the liquefaction cascade [24], which could lead to different “downstream” cleavage patterns. The role of the potential peptidases involved in the formation of the specific peptide fragments cannot be judged at present. In further experimental studies the possible involvement of exopeptidases should be addressed, which might further process the initial fragments. However, current literature is insufficient to assign the special cleavage sites within semenogelin to distinct exopeptidases [25].

Our study faces several limitations. Donation of seminal plasma for diagnostic purposes is related to several practical issues. From the present study we learned that between 30–50% of the patients are willing and able to donate ejaculate before radical prostatic surgery. However, we believe that acceptance will improve by communicating the promising results of our preliminary study.

We could partially compensate missing compliance by the inclusion of healthy volunteers and patients with chronic prostatitis. Although these cohorts enabled us to confirm our initial hypotheses that seminal plasma offers a robust source of biomarkers, they might also have introduced some degree of bias related to their age discrepancy compared to PCa and BPH groups. In addition, our cross-sectional test cohorts are relatively small and skewed. Therefore, future confirmatory studies should mind well powered, balanced, and age-matched control cohorts with clinical outcome data on PCa subtypes in follow-up. Based on the small-scale test data presented here, sample size calculations for such kind of study estimate a total sample size of 200 patients with advanced or aggressive PCa and 302 patients with localized indolent disease to demonstrate a minimal sensitivity and specificity of 70% and 80% for advanced PCa, respectively.

Although using state-of-the-art tandem mass spectrometry, we were unable to sequence all biomarker candidates. In contrast to identification of parent proteins by tryptic peptide mass fingerprinting, native peptide sequencing is limited by post-translational modifications, complicating not only peptide fragmentation, but also subsequent database searches.

## Conclusions

We were able to confirm our initial hypothesis that seminal fluid is a robust source for the identification of PCa protein maker profiles for primary diagnosis of prostate cancer. Our study involves a two-step experimental approach with independent discovery and test sets of samples in relation to post-surgery clinical reference standard. This design is in line with current guidelines for clinical proteome analysis [26]. Although our cohorts are relatively small and selected, they were appropriate to assess the feasibility of seminal profiling and to estimate the potential of seminal peptides as diagnostic biomarkers. Therefore, the present study should be understood as a very first step into the field of seminal biomarkers. Our findings warrant further confirmatory studies with enlarged unselected prospective validation cohorts to confirm and to precise the diagnostic potential of the seminal biomarker candidates and their (patho)physiological relevance.

## Materials, Patients and Methods

### Ethics Statement

The study was approved by the Ethics Committee of the University of Leipzig (Reg.No. 084-2009-20042009) and was conducted according to the principles expressed in the Declaration of Helsinki. Written informed consent was obtained from all patients.

### Study design and seminal plasma sampling

Exploitable seminal plasma samples were obtained from 70 patients with PCa, 21 patients with benign prostate hyperplasia (BPH), 25 patients with chronic prostatitis (CP) and 9 healthy controls (HC). As clinical reference standard we used a combination of histological workup of radical prostatectomy specimens for post-surgery tumour grading and staging in PCa patients and negative 10–12 needle prostate biopsy cores and/or negative prostate resection specimens in BPH patients. All patients were asked to donate seminal fluid prior to radical surgical resection of the prostate, during infertility or urological diagnostics. For biomarker discovery the available 125 samples were separated into three study arms, one for diagnostic biomarkers (study A), a second for advanced disease biomarkers with different training and test sets (study B), and biomarker stability and reproducibility (study C, Figure 1). In studies A and B, samples were either used for discovery or for performance tests, but not both. Fifty samples (22 PCa, 9 BPH, 14 CP, 5 HC) were used as training set for diagnostic biomarker discovery (Table 1A), 75 samples were included into the test set for testing diagnostic performance (48 PCa, 12 BPH, 11 CP, 4 HC, Table 1B). For advanced disease biomarker discovery we divided the available 70 PCa samples into a training set with 37 PCa samples (21 GS<7, 16 GS>7). The remaining 33 samples with GS = 7 were used as a test set (28 <pT3a “indolent”, 5 ≥pT3a “advanced”).

We compared five different approaches for assessment of risk for clinical PCa progression: based on the guidelines of the AUA [27] who adopted the D’Amico criteria [28], the National Comprehensive Cancer Network (NCCN) criteria [29], the Radiation Therapy Oncology Group (RTOG) criteria [30], the European Association of Urology (EAU) guidelines [31], and the Cancer of the Prostate Risk Assessment Score (CAPRA) score [32] (Table S4). Seminal plasma samples were internally coded and analysed in a blinded fashion (test set) after establishing biomarker profile (training set).

In order to analyze pre-analytical stability of seminal plasma obtained by this sampling protocol, a single sample of a patient harbouring PCa was thawed and prepared in two independent replicates (study C). The rest of the sample was incubated at room temperature. For six hours, every hour two replicates were prepared. All 14 prepared replicates were lyophilized shortly after preparation and re-suspended immediately before CE-MS analysis.

Analytical precision of the established SVM classifiers was assessed by applying it to 15 CE-MS data sets obtained from independent replicates of a sample of a 57 years old patient with significant BPH. Prostate volume was 120 cc and total serum PSA 4.3 ng/mL. Results were expressed as mean and standard deviation. Coefficients of variations were calculated by dividing standard deviations by the observed overall range of SVM scores [21PP from –1.50 to +1.50 (3.0 a.u.), 5PP from –4.50 to +3.0 (7.5 a.u.), and 11PP from –1.50 to +1.50 (3.0 a.u.)].

### Sample procurement and proteomic analysis

Ejaculate was collected and allowed natural liquefaction to occur by proteolysis at room temperature for 15 to 30 min. Subsequently specimens were centrifuged at 4000 rpm for 10 min to separate spermatozoa from seminal plasma. The supernatant was then aliquoted into 50 µl aliquots and deep frozen at –80°C until further processing.

### Sample preparation

Immediately before preparation, seminal plasma samples were thawed and protein concentration was adjusted to 2 mg/ml. 10 µl-replicates were lyophilized, stored at 4°C. Shortly before CE-MS analysis the lyophilized replicates were suspended in 9 µl high-performance liquid chromatography grade H_2_O and 1 µl synthetic isotope-labelled peptide stock solution (Table S5) was added [11] Isotope-labelled peptides were purchased from JPT (Berlin, Germany). One ^15^N- and five ^13^C-isotopes were incorporated into a single proline residue (ΔM = +6). The C-termini of the isotope-labelled peptides were synthesized with an amide function (ΔM = –1). Therefore, the synthetic peptides had a total mass difference of 5 atomic mass units. The injected amounts of synthetic peptides are given in (Table S5).

### CE-MS analysis

CE-MS analysis was performed as described earlier [33], [34]. By this procedure the limit of detection was ∼1 fmol. Mass resolution was above 8,000 enabling resolution of monoisotopic mass signals for z≤6. After charge deconvolution, mass accuracy was <25 ppm for monoisotopic resolution and <100 ppm for unresolved peaks (z>6).

Data sets were accepted only if the following quality control criteria were met: A minimum of 1000 peptides/proteins must be detected with a minimum MS resolution of 8,000 (required resolution to resolve ion signals with z = 6) in a minimum migration time interval (the time window, in which separated peptides can be detected) of 10 minutes (mean number time interval minus one standard deviation). After calibration, the mean deviation of migration time (compared to reference standards) must be below 0.30 minutes.

### Data processing

Mass spectral ion peaks representing identical molecules at different charge states were deconvoluted into single masses using MosaiquesVisu software (www.proteomiques.com) [35]. For noise filtering, signals with z>1 observed in a minimum of 3 consecutive spectra with a signal-to-noise ratio of at least 4 were considered. MosaiquesVisu employs a probabilistic clustering algorithm and uses both isotopic distribution (for z≤6) as well as conjugated masses for charge-state determination of peptides/proteins. The resulting peak list characterizes each polypeptide by its mass and its migration time. After charge deconvolution, mass accuracy was <25 ppm for monoisotopic resolution and <100 ppm for unresolved peaks (z>6). First we used 14 synthetic isotope labeled peptides for data normalization. These peptides were added to samples immediately before CE-MS analysis (Table S5). Secondly, we calibrated the CE-MS data utilizing 287 reference mass data points and migration time data points by applying global and local linear regression, respectively. Ion signal intensity (amplitude) was normalized relative to 46 reference signals of highly abundant peptides using local linear regression (Table S1). The obtained peak list characterizes each polypeptide by its calibrated molecular mass [Da], calibrated CE migration time [min] and normalized signal intensity. All detected peptides were deposited, matched, and annotated in a Microsoft SQL database allowing further statistical analysis. For clustering, peptides in different samples were considered identical, if mass deviation was <50 ppm for small (<4,000 Da) or 75 ppm for larger peptides. Due to analyte diffusion effect, CE peak widths increase with CE migration time. For data clustering this effect was considered by linearly increasing cluster widths over the entire electropherogram (19 min to 45 min) from 2-5%.

After data normalization, all detected peptides were deposited, matched, and annotated in a Microsoft SQL database. As previously described for urine [36], [37], several annotated peptides appear sporadically, being observed in only one or a few samples. To eliminate such peptides of apparently low significance, only those peptides detected in more than 6 of the seminal plasma samples in at least one group (samples from patients with same disease) were further investigated. Applying these limits, a total of 1,784 relevant native peptides were clustered with a mass range from 802.4 Da to 15,701.8 Da.

### Descriptive Statistical analysis

Estimates of sensitivity and specificity were calculated based on tabulating the number of correctly classified samples. Confidence intervals (95% CI) were based on binomial calculations performed with MedCalc version 8.1.1.0 (MedCalc Software, Belgium, www.medcalc.be). The ROC plot was evaluated, as it provides a single measure of overall accuracy that is not dependent upon a particular threshold [38].

### Differential statistical analysis

For statistical differential analysis we set a frequency threshold of 60% for markers to be deemed valid in one of the considered groups in order to be included in downstream analysis. Adjustments for multiple testing [39] were done using the base 10 logarithm transformed intensities and the Gaussian approximation to the t-distribution. For multiple testing corrections, p-values were corrected using the false discovery rate procedure introduced by Benjamini and Hochberg, which conserves sufficient statistical power of looking for biomarkers that are differentially expressed between two samples when subjected to two different treatments, such as disease/no disease [40]. Proteins that were detected in a diagnostic group of patients in at least 60% of samples were considered. The test was implemented as macros in SAS (www.sas.com) and are part of the multitest R-package www.bioconductor.org [41].

### Classification

MosaCluster (version 1.7.0) was developed for the discrimination between different patient groups. This software tool allows the classification of samples in the high-dimensional parameter space by using support vector machine (SVM) learning. For this purpose, MosaCluster generates polypeptide models, which rely on polypeptides displaying statistically significant differences when comparing data from patients with a specific disease to controls or other diseases, respectively. Each of these polypeptides allegorizes one dimension in the n-dimensional parameter space [36], [42]–[44]. SVM view a data point (probands urine sample) as a p-dimensional vector (p numbers of protein used), and they attempt to separate them with a (p-1) dimensional hyperplane. There are many hyperplanes that might classify the data. However, maximum separation (margin) between the two classes is of additional interest, and therefore, the hyperplane with the maximal distance from the hyperplane to the nearest data point is selected. Therefore, all marker proteins are used without any weighting to build up the n-dimensional classification space and to display the data set in the classification space. Classification itself is performed by determining the Euclidian distance of the data set to the n-1 dimensional maximal margin hyperplane (absolute value of the normal vector) and the direction of the vector (class 1 or class 2).

### Diagnostic cut-offs

For all biomarker patterns the threshold (cut-off) indicating the transition from “negative” to “positive” was established based on the classification results of the training set, considering analytical variation of the system: This ensures a less than 15% chance that a measurement with a true classification result of the threshold value would give a false result above the cut-off. For 21PP the analytical precision revealed a standard deviation SD (precision) of approximately 0.30 a.u. The final cut-off was calculated as 0.30–1SD = 0.00 a.u. This cut-off was applied to the 75 samples of the test set. Values below 0.00 were considered negative, values ≥0.00 positive. For 5PP the final cut-off was calculated as 1.00+1SD = 1.48 a.u. Values below 1.48 were considered negative for BPH, values ≥1.48 positive. For 11PP the final cut-off was calculated as 0.01+1SD = 0.30 a.u. This cut-off was applied to the 33 samples of the test set. Values below 0.30 were considered as indolent disease, values ≥0.30 as advanced disease.

### Sequencing of peptides

Native peptides from seminal plasma were sequenced using LC-MS/MS analysis. MS/MS experiments were performed using higher energy collision dissociation (HCD) or electron transfer dissociation (ETD) [12]–[14]. Peptides were separated on a Dionex Ultimate 3000 RSLS nano flow system (Dionex, Camberly UK) and introduced into an LTQ Orbitrap hybrid mass spectrometer (Thermo Fisher Scientific, Bremen, Germany) via nano-flow ESI, as described in Metzger et al. [45]. Data files were searched against the IPI human non-redundant database using the Open Mass Spectrometry Search Algorithm (OMSSA, http://pubchem.ncbi.nlm.nih.gov/omssa), with an e-value cut-off of 0.05 without any enzyme specificity. No fixed modification was selected, and oxidation of methionine were set as variable modifications. Accepted parent ion mass deviation was 10 ppm; accepted fragment ion mass deviation was 0.05 Da (for HCD) or 0.5 Da (for ETD). For further validation of obtained peptide identifications, the strict correlation between peptide charge at the working pH of 2 and CE-migration time was utilized to minimize false-positive identification rates [46]: Calculated CE-migration time of the sequence candidate based on its peptide sequence (number of basic amino acids) was compared to the experimental migration time. Peptides were accepted with a mass deviation below ±80 ppm and a CE-migration time deviation below ±2 min.

## Supporting Information

Table S1 **CE-MS data sets.** For detected peptides identification tag, calibrated mass in Da and migration time in min are given. For all 125 patient data sets normalized signal amplitude are listed, whereas “0” denotes undetected or missing values.
(XLS)

Table S2 **Internal references.** 46 seminal polypeptides were used as internal references for signal amplitude normalization. ID: polypeptide identifier annotated by the SQL database (ID), Amp: Signal amplitude, CV: coefficient of variation of signal amplitudes.
(DOC)

Table S3 **Seminal peptide sequence data.** Tandem mass spectrometry identified 141 native seminal peptides representing 47 different parental proteins. Fifthy-nine percent were fragments of semenogelin-1 or -2, by far the most abundant peptides of the low molecular weight seminal proteome. ID: polypeptide identifier annotated by the SQL database (ID); Theo. Mass: theoretical mass of the peptide sequence; delta CE/MS-M: Mass difference between CE-MS experimental and theoretical mass normalized to theoretical mass in parts per million [ppm]; delta MS/MS-M: Mass difference between MS/MS experimental and theoretical mass normalized to theoretical mass in parts per million [ppm]; m: oxidized Methionine; CE/MS-Mass: CE-MS experimental mass in Dalton [Da]; MS/MS-Mass [Da]: MS/MS experimental mass in Dalton [Da]; E-value: Score used by OMSSA to rank hits for a given MS/MS-spectrum.
(XLS)

Table S4 **Risk assessment classification systems.** In clinical practice various classification systems are used to estimate risk for prostate cancer progression. Therefore, we compared the performance of our biomarkers to five commonly used systems, namely AUA guidelines who adopted the D’Amico criteria, the National Comprehensive Cancer Network (NCCN) criteria, the Radiation Therapy Oncology Group (RTOG) criteria, the European Association of Urology (EAU) guidelines, and the Cancer of the Prostate Risk Assessment Score (CAPRA) score.
(DOC)

Table S5 **Characteristics of synthetics peptide used for pre-calibration of seminal plasma samples.** Isotope labelled proline residues are marked in bold italics. The amount of each synthetic peptide added to the samples and averaged MS-detected intensity are given.
(DOC)
