# Supplementary material for: Seminal Plasma as a Source of Prostate Cancer Peptide Biomarker Candidates for Detection of Indolent and Advanced Disease
Source: PLoS One. 2013 Jun 24;8(6):e67514. doi: 10.1371/journal.pone.0067514 (PMC3691205; doi:10.1371/journal.pone.0067514)
Supplement: Table S2 — Internal references. 46 seminal polypeptides were used as internal references for signal amplitude normalization. ID: polypeptide identifier annotated by the SQL database (ID), Amp: Signal amplitude, CV: coefficient of variation of signal amplitudes. (DOC) [file pone.0067514.s002.doc]

**Supplementary table 2:** 46 Seminal polypeptides used as internal references for signal amplitude normalization.

| **Calibrant ID** | **Mass (Da)** | **CE time (min)** | **Mean Amp** | **CV [%]** |
| --- | --- | --- | --- | --- |
| 3531 | 1097.56 | 24.8 | 75 | 44 |
| 4226 | 1158.52 | 25.9 | 211 | 58 |
| 4256 | 1160.52 | 27.8 | 1971 | 73 |
| 5058 | 1225.66 | 21.6 | 13897 | 83 |
| 5090 | 1228.64 | 25.8 | 146 | 56 |
| 5286 | 1242.66 | 26.3 | 139 | 55 |
| 5659 | 1277.56 | 29.5 | 1646 | 90 |
| 6192 | 1326.67 | 20.4 | 864 | 67 |
| 6199 | 1327.63 | 27.2 | 1821 | 32 |
| 6291 | 1334.58 | 29.6 | 10471 | 89 |
| 6382 | 1341.63 | 27.4 | 441 | 38 |
| 7090 | 1403.67 | 23.3 | 13906 | 75 |
| 7447 | 1438.63 | 27.8 | 860 | 58 |
| 7630 | 1456.64 | 28.4 | 3940 | 28 |
| 7665 | 1459.76 | 22.4 | 5079 | 74 |
| 8243 | 1513.66 | 28.9 | 289 | 52 |
| 8406 | 1531.83 | 22.2 | 1439 | 64 |
| 8950 | 1589.83 | 17.6 | 8014 | 76 |
| 8974 | 1591.84 | 28.4 | 198 | 37 |
| 9372 | 1634.89 | 24.2 | 13804 | 84 |
| 9422 | 1638.85 | 20.6 | 34058 | 60 |
| 9509 | 1647.84 | 17.7 | 2771 | 90 |
| 9719 | 1669.79 | 23.4 | 175 | 63 |
| 10100 | 1705.97 | 23.0 | 162 | 55 |
| 10422 | 1740.83 | 20.7 | 593 | 50 |
| 10485 | 1747.97 | 23.4 | 6484 | 51 |
| 10551 | 1754.82 | 30.2 | 215 | 45 |
| 10801 | 1782.84 | 23.8 | 447 | 53 |
| 10966 | 1801.95 | 24.3 | 2111 | 79 |
| 11950 | 1935.05 | 23.9 | 2426 | 29 |
| 12347 | 1996.94 | 24.6 | 1252 | 68 |
| 12544 | 2026.93 | 25.8 | 837 | 52 |
| 13305 | 2140.97 | 26.3 | 1612 | 59 |
| 13563 | 2178.14 | 24.9 | 345 | 38 |
| 13595 | 2183.97 | 25.7 | 871 | 58 |
| 13645 | 2192.16 | 25.0 | 620 | 39 |
| 13674 | 2196.98 | 26.5 | 185 | 54 |
| 14148 | 2263.26 | 21.9 | 2248 | 74 |
| 14177 | 2266.16 | 25.4 | 794 | 88 |
| 14387 | 2294.24 | 21.8 | 579 | 56 |
| 14661 | 2337.27 | 22.0 | 436 | 48 |
| 15338 | 2444.30 | 19.9 | 6026 | 87 |
| 15377 | 2450.35 | 22.3 | 1514 | 50 |
| 16361 | 2607.29 | 20.9 | 18429 | 65 |
| 19667 | 3135.65 | 22.1 | 17883 | 57 |
| 25626 | 4420.26 | 19.2 | 14056 | 63 |

ID: polypeptide identifier annotated by the SQL database (ID)

Amp: Signal amplitude

CV: coefficient of variation
