# Supplementary material for: Seminal Plasma as a Source of Prostate Cancer Peptide Biomarker Candidates for Detection of Indolent and Advanced Disease
Source: PLoS One. 2013 Jun 24;8(6):e67514. doi: 10.1371/journal.pone.0067514 (PMC3691205; doi:10.1371/journal.pone.0067514)
Supplement: Table S4 — Risk assessment classification systems. In clinical practice various classification systems are used to estimate risk for prostate cancer progression. Therefore, we compared the performance of our biomarkers to five commonly used systems, namely AUA guidelines who adopted the D’Amico criteria, the National Comprehensive Cancer Network (NCCN) criteria, the Radiation Therapy Oncology Group (RTOG) criteria, the European Association of Urology (EAU) guidelines, and the Cancer of the Prostate Risk Assessment Score (CAPRA) score. (DOC) [file pone.0067514.s004.doc]

**Supplemental Table 4:** Risk assessment classification systems

|  | **risk group** | **clinical stage**  **(alternative)** | **biopsy  Gleason Score**  **(alternative)** | **PSA level**  **[ng/ml]** |  |
| --- | --- | --- | --- | --- | --- |
| **D’Amico / AUA** | low | ≤ T2a | and ≤ 6 | and ≤10 |  |
|  | intermediate | T2b | or 7 | or >10 and ≤20 |  |
|  | high | ≥ T2c | or ≥ 8 | or > 20 |  |
| **NCCN** | low | ≤ T2a | ≤ 6 | < 10 |  |
|  | high | ≥ T3a  (T2b7c) | or ≥ 8  (7) | or ≥ 20  (≤ 20) | or any two of the alternatives |
| **EAU** | low | ≤ T2c | ≤ 7 | < 20 |  |
|  | high | ≥ T3a | or ≥ 8 | or ≥ 20 |  |
| **RTOG** | low | ≤ T2 | ≤ 6 |  |  |
|  | intermediate | ≤ T2 (T3) | 7 (≤ 6) |  |  |
|  | high | ≤ T3 (T3) | and ≥ 8 (7) |  |  |
| **CAPRA** | low | ≤ T2a | and GS pattern <4 | and < 10 |  |
|  | intermediate | T2b-c | 7 (<7 with secondary GS pattern ≥ 4) | and > 10 and ≤ 20 |  |
|  | high | ≥ T3a | ≥ 8 (primary GS pattern ≥4) | and > 20 |  |
