# Supplementary material for: Seminal Plasma as a Source of Prostate Cancer Peptide Biomarker Candidates for Detection of Indolent and Advanced Disease
Source: PLoS One. 2013 Jun 24;8(6):e67514. doi: 10.1371/journal.pone.0067514 (PMC3691205; doi:10.1371/journal.pone.0067514)
Supplement: Table S5 — Characteristics of synthetics peptide used for pre-calibration of seminal plasma samples. Isotope labelled proline residues are marked in bold italics. The amount of each synthetic peptide added to the samples and averaged MS-detected intensity are given. (DOC) [file pone.0067514.s005.doc]

**Supplemental Table 5**: Characteristics of the synthetics peptide used for pre-calibration of seminal plasma samples.

| **mass [Da]** | **CE-time [min]** | **sequence information*** | **parent protein** | **amount injected [ng]**** | **Mean intensity**** |
| --- | --- | --- | --- | --- | --- |
| 1101.50 | 25.4 | ApGDRGEpG***P***p | Collagen alpha-1 (I) chain | 2.651 | 6214±2684 |
| 1270.61 | 26.5 | SpG***P***DGKTGPpGPA | Collagen alpha-1 (I) chain | 6.000 | 19684±13833 |
| 1584.74 | 29.2 | SpGSpG***P***DGKTGPPGpAG | Collagen alpha-1 (I) chain | 0.506 | 3068±1328 |
| 1697.79 | 29.8 | ***P***pGEAGKpGEQGVPGDLG | Collagen alpha-1 (I) chain | 0.246 | 2885±1288 |
| 1948.05 | 24.2 | EAI***P***MSIPPEVKFNKPF | Alpha-1-antitrypsin | 6.050 | 155318±58265 |
| 2068.96 | 21.6 | NGDDGEAGKpGRpGERG***P***pGP | Collagen alpha-1 (I) chain | 1.865 | 9426±4956 |
| 2083.96 | 26.1 | DAGApGApGGKGDAGApGERG***P***pG | Collagen alpha-1 (III) chain | 3.693 | 12488±6890 |
| 2164.04 | 32.6 | AG***P***pGEAGKpGEQGVpGDLGAPGP | Collagen alpha-1 (I) chain | 0.358 | 4357±2661 |
| 2180.03 | 32.7 | AG***P***pGEAGKpGEQGVpGDLGApGP | Collagen alpha-1 (I) chain | 1.008 | 8291±4776 |
| 2297.04 | 26.8 | ADGQpGAKGEpGDAGAKGDAG***P***pGPA | Collagen alpha-1 (I) chain | 1.735 | 6062±3106 |
| 2382.12 | 20.7 | GKNGDDGEAGKpGRpGERG***P***pGPQ | Collagen alpha-1 (I) chain | 0.969 | 2893±1989 |
| 2394.27 | 22.1 | MIEQNTKS***P***LFMGKVVNPTQK | Alpha-1-antitrypsin | 3.413 | 78365±34918 |
| 2476.18 | 34.4 | TG***P***IGPpGPAGApGDKGESGPSGPAGPTG | Collagen alpha-1 (I) chain | 0.215 | 724±465 |
| 3113.45 | 30.9 | ADGQpGAKGEpGDAGAKGDAGpPG***P***AGPAGPPGpIG | Collagen alpha-1 (I) chain | 0.271 | 706±413 |

* Isotope labelled proline residues are marked in bold italics

** The amount of each synthetic peptide added to the samples and averaged MS-detected intensity
